# Hereditary diffuse gastric cancer: a case report

**DOI:** 10.3389/fonc.2025.1634470

**Published:** 2025-11-25

**Authors:** Lili Li, Youzhi An, Yanjun Wang

**Affiliations:** 1 Medical Oncology, The Second People’s Hospital of Liaocheng, The Second Hospital of Liaocheng Affiliated to Shandong First Medical University, Lingqing, Shandong, China; 2 Second Department of Spinal Surgery, The Second People’s Hospital of Liaocheng, The Second Hospital of Liaocheng Affiliated to Shandong First Medical University, Linqing, Shandong, China

**Keywords:** hereditary diffuse gastric cancer, *CDH1*, signet-ring cell carcinoma, E-cadherin, hereditary gastric cancer

## Abstract

Hereditary diffuse gastric cancer (HDGC) is an autosomal dominant genetic syndrome characterized by distinct clinical and genetic features. It exhibits low clinical incidence, familial clustering, early onset, insidious progression, and challenges in early diagnosis. In addition, HDGC is marked by poor tumor differentiation, high malignancy, specific gene mutations, frequent occurrence of extra-gastric tumors, and a high risk of inheritance, which poses significant challenges to clinical medicine, medical genetics, and reproductive medicine. In this study, we have reported a case of HDGC in a three-generation Chinese family of four individuals. By integrating clinical, pathological, imaging, genetic mutation, family history, diagnostic and treatment process, and the survival outcome data, it fully demonstrates the clinical heterogeneity of HDGC and the considerable dilemmas encountered in its management. These findings together provide valuable insights into the clinical diagnosis and treatment of related cases, literature research, as well as the management of cancer-related genetic diseases and reproductive health.

## Introduction

The 2020 Global Cancer Statistics reported 1.09 million new cases of gastric cancer (GC), making it the fifth most frequently occurring malignant tumor. With the report of 770,000 deaths associated with GC, it now ranks fourth in terms of the mortality rate (1). China has a high incidence rate of GC. In 2020, 480,000 new cases and 370,000 deaths due to GC were reported in China alone, and, in terms of the associated mortality, GC ranked third among malignant tumors (2).

GC manifests as sporadic GC, familial GC (FGC), and hereditary GC (HGC); most cases of GC are sporadic, followed by familial aggregation cases occurring in 5–10% of GC patients (3). Genetic predisposition cases occur in 1–3% of all GC patients (3).

Familial aggregation GC refers to a clustered incidence of GC in a family, often attributable to the common living environment, diet, some accidental factors, or even common genetic factors. As such, the category of familial aggregation GC should include familial hereditary GC. Familial hereditary GC is an autosomal dominant genetic disease (or hereditary tumor syndrome), most of which involves clear pathogenic gene mutations inherited from the family, which mainly includes the following three syndromes: hereditary diffuse GC (HDGC), gastric adenocarcinoma and proximal polyposis of the stomach (GAPPS), and familial intestinal GC (FIGC) (3).

HDGC is currently defined as the presence of a pathogenic germline *CDH1* or *CTNNA1* variant in either an isolated individual with diffuse GC (DGC) or in a family with one or more DGC cases among first- or second-degree relatives (4). HDGC is an autosomal dominant genetic disease, and *CDH1* encoding E-cadherin and *CTNNA1* encoding alpha-E-catenin have been identified as susceptibility genes (3).

HDGC is often caused due to inactivation mutations of the tumor suppressor gene *CDH1*, and a small portion of the HDGC families exhibit *CTNNA1* abnormalities (3). It has been reported that the incidence of *CDH1* mutation in HDGC patients varies across countries and races, and the mutation rate is inversely proportional to the incidence rate of GC (3). In Canada, the United States, and the United Kingdom, the incidence rate of GC is the lowest, whereas the incidence of *CDH1* mutation in HDGC patients is the highest (up to 40–52.6%). In Germany and other countries with a medium incidence rate of GC, the mutation rate of *CDH1* is moderate (approximately 25%). In Portugal, Italy, Japan, and other countries that have the highest incidence rate of GC, the incidence of *CDH1* mutation is only 15.4–22.2%. There are currently no reports on the frequency of *CDH1* mutations among Chinese HDGC patients, although the incidence of *CDH1* mutations among Chinese GC patients is <1% (3). Most of the confirmed cases of HDGC are caused by *CDH1* germline mutations (30–50%) and a few (1.4%) by *CTNNA1* germline mutations (5).

*CDH1* is located on human chromosome 16q22.1 and contains 16 exons (2). The *CDH1* encodes the calcium-dependent cell adhesion protein E-cadherin, which plays a major role in cell adhesion and tumor suppression (2). The loss of E-cadherin function can affect epithelial cell proliferation, cell mucosa and polarity, and cell migration, resulting in cell invasion and metastasis (2). In the *CDH1* mutation-negative HDGC family, *CTNNA1* displays a mutation (2). The alpha-E-catenin protein encoded by *CTNNA1* plays an important role in cell adhesion and becomes a tumor suppressor gene after connecting with the plasma membrane calcium-binding proteins and intracellular actin filaments (2). Considering the involvement of CTNNA1 in intracellular adhesion, this loss-of-function mutation may be similar to the *CDH1* mutation (6). *CTNNA1* is the only gene that is specifically prone to DGC, but not to intestinal-type GC (7). The cumulative risk of developing GC in individuals carrying *CDH1* mutations up to the age of 80 years is approximately 70% (males) and 56% (females) (7), whereas the risk of developing DGC in individuals carrying *CTNNA1* mutations up to the age of 80 years is 49–57% (8).

There are very few case reports on hereditary diffuse gastric cancer (HDGC) among Chinese individuals with CDH1 mutations. This study addresses this literature gap by providing a comprehensive analysis of a clinical case, supplemented with a review of relevant publications. It highlights the key characteristics of HDGC, including early disease onset (age 17 years in the proband), high-grade malignancy, poor prognosis, and significant familial aggregation with transgenerational inheritance. Notably, the youngest affected individual in this pedigree presented with congenital absence of mandibular incisors and was found to harbor additional genetic mutations alongside the pathogenic CDH1 variant, which was associated with a highly aggressive tumor phenotype. This particular finding, a co-mutational profile leading to enhanced clinical aggressiveness, has not been previously documented in any HDGC case and merits further investigation.

## Case presentation

### Case III-1 (the proband)

**Figure 1:**
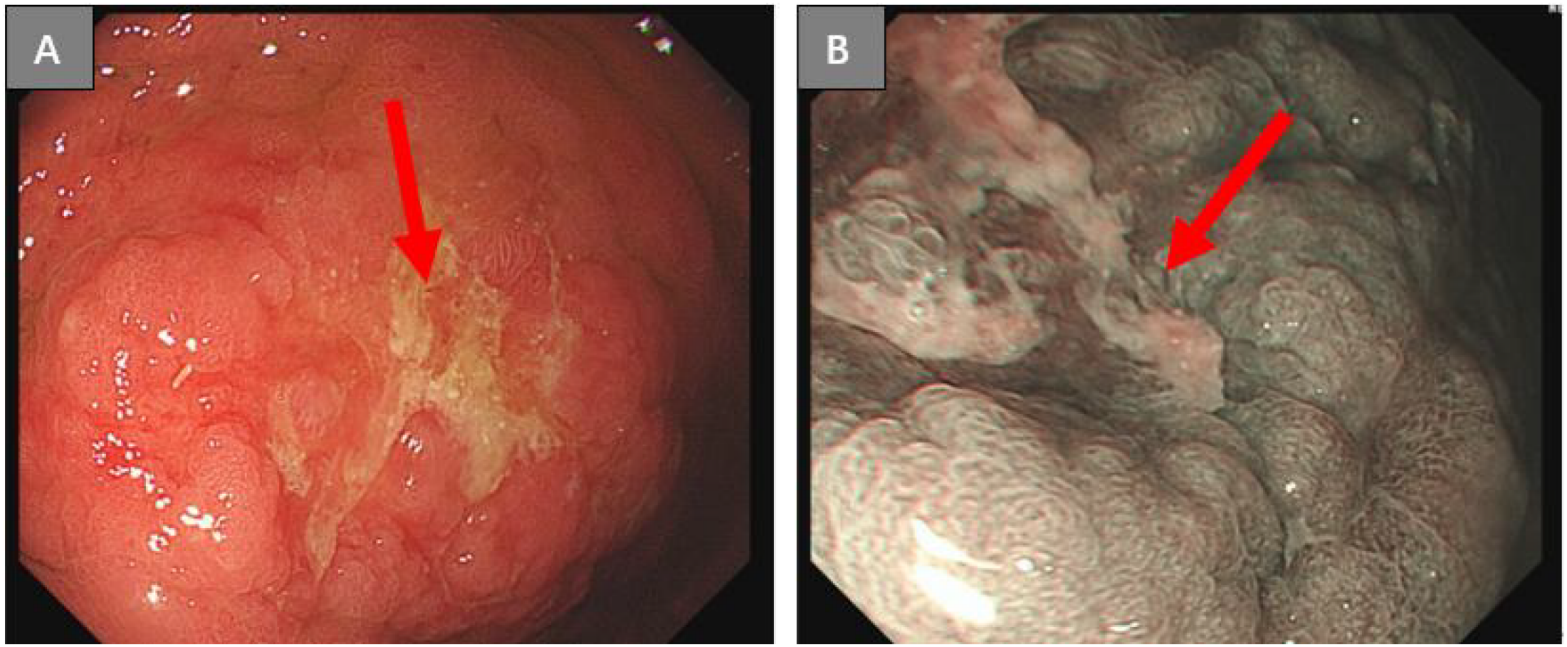
Gastroscopy image of the proband patient: A 1.0 cm × 1.5 cm superficial irregular ulcer was observed on the greater curvature side of the gastric body, covered with yellowish-white fur, accompanied by surrounding mucosal edema, and hyperplasia **(A)** under NBI, the glandular ducts were observed to be disordered **(B)**.

**Figure 2:**
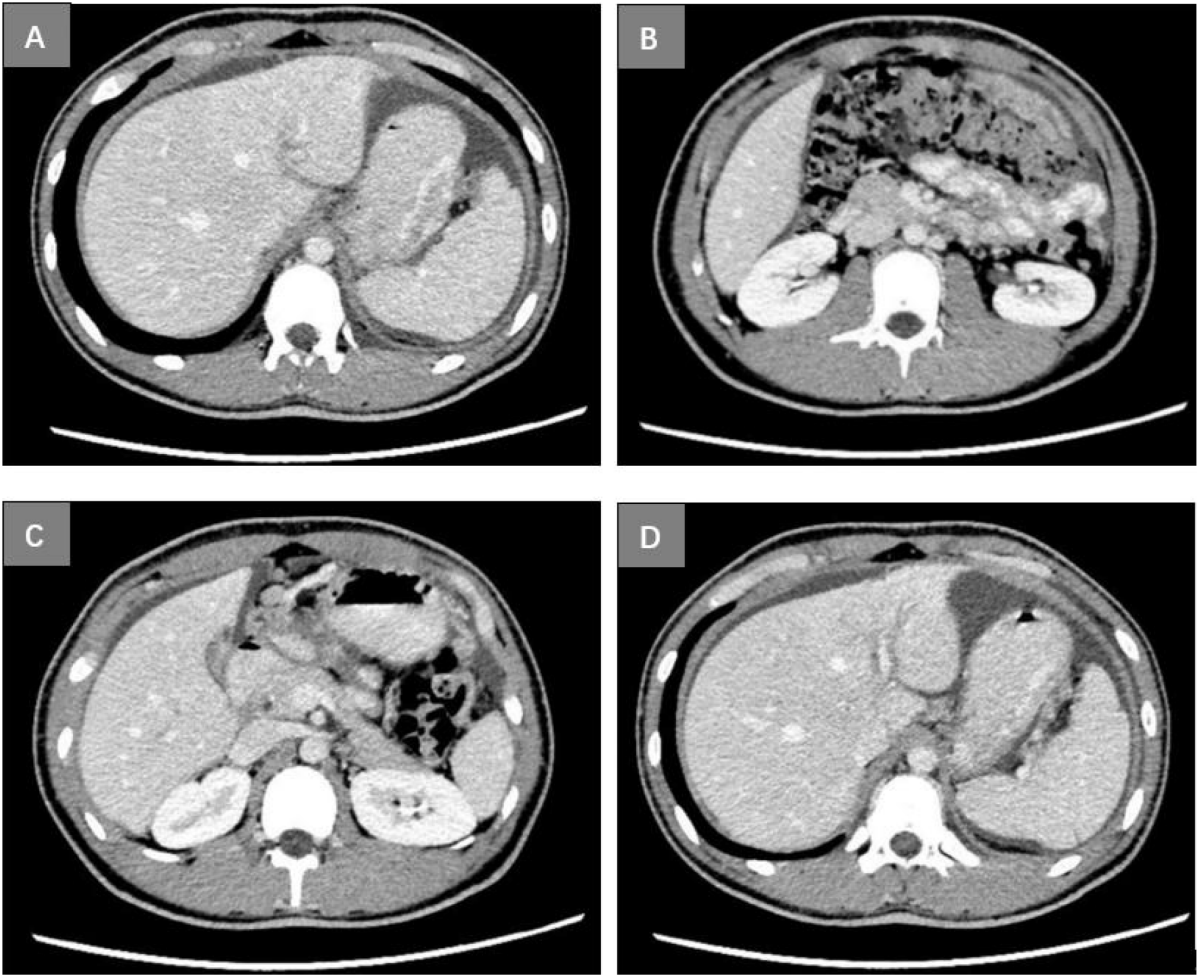
The CT image of the proband patient: the thickening of the gastric wall on the greater curvature side, with a thicker area measuring approximately 2.1 cm, and uneven enhancement on the contrast-enhanced scan **(A)** nodular or pancake-like thickening and enhancement of the peritoneum **(B)** one enlarged lymph node can be noticed around the stomach, with enhancement visible on the contrast-enhanced scan, measuring approximately 1.0 cm in diameter **(C)** free fluid-like density shadow was observed in the abdominal cavity **(D)**.

**Figure 3:**
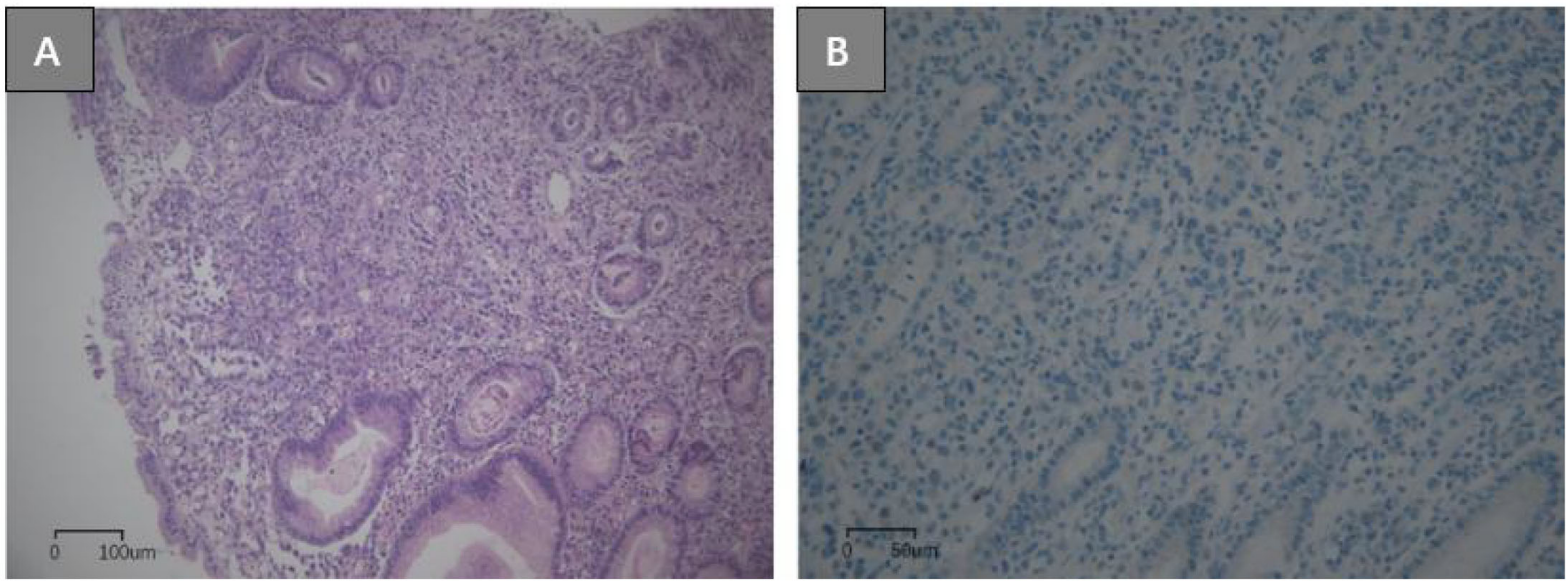
Pathological images of gastroscopy biopsy of the proband patient: (gastric body) Some tumor cells in the mucosa exhibited poor adhesion, with a patchy arrangement. There is obvious cellular atypia, with polygonal cells, large and deeply stained nuclei, visible mitotic figures, and focal tumor cell nuclei pushed to one side by mucus, resembling signet ring cells. Some tumor cells are arranged in glandular tubes with irregular shapes, resembling disorder, and distributed unevenly with poorly differentiated cancer components. A small amount of lymphocytes and monocytes can be seen infiltrating the tumor stroma (hematoxylin eosin staining ×100) **(A)** HER-2 immunohistochemistry staining: 0, no staining (immunohistochemistry staining ×200) **(B)**.

A 17-year-old boy suffering from congenital loss of lower incisors was admitted to Linqing People’s Hospital, China, for upper abdominal pain and heartburn for more than 1 month on May 15, 2022. His gastroscopy examination revealed a shallow, irregular ulcer measuring 1.0 × 1.5 cm on the greater curvature side of the stomach, covered with yellow and white lichen, displaying edema and hyperplasia of the surrounding mucosa. Under narrow-band imaging (NBI), a disorder was recorded in the arrangement of glandular ducts (**Figure 1**). Microscopic examination of the gastric biopsy revealed a gastric ulcer, with findings consistent with poorly differentiated adenocarcinoma and signet ring cell carcinoma. On May 18, 2022, the patient visited the Second People’s Hospital of Liaocheng for diagnosis and treatment purposes. His (Chest+Upper Abdominal) Plain Scan+Enhanced Computed Tomography (CT) revealed thickening of the gastric wall on the greater curvature side, which was consistent with changes in the tumor lesions (approximately 2.1 cm at the thicker portion). An enlarged lymph node, measuring approximately 1 cm (diameter), was detected around the stomach, with enhancement recorded on contrast scan. Nodular or pancake-like thickening of the peritoneum and peritoneal metastasis suggested abdominal dropsy (**Figure 2**). Pathological consultation through gastric body biopsy mainly revealed poorly differentiated adenocarcinoma with some signet ring cell carcinoma (**Figure 3**). The immunohistochemistry results were as follows: HER-2 (-) (**Figure 3**), PMS2 (+), MLH1 (+), MSH2 (+), MSH6 (+).

With reference to the AJCC/UICC 8^th^ edition TNM staging of GC, the clinical staging was identified as stage IV poorly differentiated adenocarcinoma of the gastric body (cT4aN1M1). The patient’s height was 172 cm, weight 57 kg, body surface area 1.69 m^2^, and an ECOG score of 0. According to the 2021 Chinese Society of Clinical Oncology (CSCO) Guidelines for the Diagnosis and Treatment of Gastric Cancer, the first-line treatment plan for advanced metastatic GC with Her-2 negative was strategized as follows: in the Grade I recommended plan, the combination of oxaliplatin and tegafur gimeracil oteracil potassium capsule (i.e., SOX) is classified as Class 1A. However, at that time point, considering that the patient was a minor who was scheduled to take the Chinese college entrance examination, his family was hesitant about chemotherapy, and to ensure the treatment’s efficacy and reduce any side effects caused by chemotherapy drugs (such as gastrointestinal reactions), reduced chemotherapy was administered to gradually increase the patient’s tolerance and compliance with chemotherapy.

On May 23, 2022, and then on June 13, 2022, SOX was administered for 2 cycles. On June 30, 2022, the patient visited the Cancer Hospital Chinese Academy of Medical Sciences, and his chest and whole abdomen-enhanced CT demonstrated poor filling of the gastric cavity, uneven enhancement of the gastric wall (approximately 2.7 cm thick), peritoneal thickening, and massive ascites, suggestive of metastasis. On July 1, 2022, gastroscopy revealed a swollen and raised local gastric wall at the junction of the gastric fundus with rough and eroded surface mucosa, indicating residual tumor lesions. The patient had not undergone endoscopic treatment. On July 7, 2022, the patient visited the Department of Oncology at the Cancer Hospital, Chinese Academy of Medical Sciences, and was recommended the following: the patient was young and had suffered from congenital loss of lower incisors; accordingly, genetic testing was recommended to confirm the germline and systemic mutations. The patient’s chemotherapy tolerance was acceptable, and, in the later stage of the disease, a biweekly regimen of albumin paclitaxel, oxaliplatin, and tegafur gimeracil oteracil potassium capsule was considered for treatment. The treatment plan was adjusted based on the results of genetic testing.

On July 10, 2022, the patient presented the consultation opinion from the aforementioned hospital to the Oncology Department of The Second People’s Hospital of Liaocheng. His ultrasound examination showed abdominal fluid accumulation (patchy fluid-like dark areas around the liver, spleen, and lower abdomen, with the deepest point detected in the left iliac fossa and a maximum depth of 6.0 cm). Ascites are not suitable for puncture drainage. Therefore, on July 10, 2022, intravenous chemotherapy was administered for one cycle as per the following regimen: albumin paclitaxel, oxaliplatin, and tegafur gimeracil oteracil potassium capsule.

On July 14, 2022, the genetic testing results from the Cancer Hospital Chinese Academy of Medical Sciences, indicated no mutations in *NRAS/PIK3CA/BRAF*, but mutations in exon 9 of *NRG1*, exon 6 of *TP53*, and germline mutations in *CDH1*, pMMR, and PD-L1 CPS <1. Based on these results, it was recommended that, if the patient cannot tolerate albumin paclitaxel chemotherapy, intraperitoneal chemotherapy(albumin paclitaxel, bevacizumab, along with 5-fu and interleukin-2) could be applied. His family believed that, as the previous treatment yielded poor outcomes, they did not prefer intravenous chemotherapy.

On August 11, 2022, the patient was admitted to The Second People’s Hospital of Liaocheng for abdominal distension and discomfort. At this time point, the patient’s ECOG score was 1. Often, after eating, the patient felt abdominal distension and discomfort, and his weight was reduced by 2 kg. His albumin level was 38.0 g/L, and ascites ultrasound revealed a large amount of ascites (the deepest visible liquid dark area in the abdominal cavity was located in the lower-right abdomen, with a maximum depth of approximately 9.6 cm). Ascites increased by 3.6 cm when compared to that last observed, indicating progression. On August 13, 2022, the patient underwent abdominal puncture drainage, and 5900 mL of bloody ascites was drained. On August 16, 2022, cytological examination of the ascitic fluid revealed small amounts of scattered heterologous cells. On August 17, 2022, an ascitic fluid cytology examination revealed a small amount of atypical cells, and, when considered in combination with his medical history, indicated a tendency toward poorly differentiated adenocarcinoma. The abdominal drainage relieved the patient’s abdominal distension to an extent, but the patient continued to refuse intravenous chemotherapy or intraperitoneal infusion treatment. Thus, on August 17, 2022, the patient was provided the option to remove the abdominal drainage tube and was discharged.

After his discharge, the patient’s mother re-visited the Cancer Hospital Chinese Academy of Medical Sciences, who was recommended that the patient should tie an abdominal belt after ascites drainage, and, if the patient’s condition allowed, an intraperitoneal infusion therapy with albumin paclitaxel, and bevacizumab should be administered. Accordingly, on August 24, 2022, the patient was admitted to the Oncology Department of the Second People’s Hospital of Liaocheng. On August 25, 2022, abdominal puncture and catheterization were performed for drainage. Considering that the cytology examination of the patient’s ascites displayed atypical cells, which tended to be poorly differentiated adenocarcinoma. The initial imaging examination detected peritoneal metastasis in the light of relevant guidelines, literature, and the diagnosis and treatment opinions of a superior hospital. Accordingly, with the consent of the patient’s family, the patient underwent a single session of intraperitoneal infusion therapy on August 28, 2022. No obvious side effects were detected after this therapy. The patient’s abdominal drainage tube was removed, and he was discharged on August 28, 2022. Unfortunately, on the same day after his discharge, the patient died due to respiratory cardiac arrest.

### Case II-1

The proband’s mother, a 45-year-old, visited the Cancer Hospital of the Chinese Academy of Medical Sciences on February 17, 2023, for tumor genetic susceptibility gene testing: a heterozygous pathogenic mutation in exon 12 c.1921C>T (p.G1n641 *) of *CDH1* was detected. On March 24, 2023, she underwent a gastroscopy at the Second People’s Hospital of Liaocheng, which revealed a needle-like necrotic lesion at the junction of the greater curvature of the stomach and the gastric antrum, with a soft texture. Two biopsies were taken at this site. Pathological diagnosis revealed signet ring cell carcinoma at the junction of the greater curvature of the gastric body and the antrum. On May 24, 2023, she underwent endoscopic ESD surgery at the Cancer Hospital Chinese Academy of Medical Sciences. After the surgery, she underwent regular follow-up gastroscopy, and no recurrence or metastasis was detected.

### Case II-3

The younger sister of the proband’s mother was identified with a GC during a physical examination in the same year, although the specific time remains unknown. Accordingly, she underwent “radical gastrectomy” for GC, and the postoperative pathology was “signet ring cell carcinoma.” Genetic testing revealed “*CDH1* germline pathogenic mutation,” but the detailed results of postoperative pathology and gene mutation remain unknown.

### Case I-1

The proband’s grandmother, aged 76 years, visited the Second People’s Hospital of Liaocheng for “abdominal pain for more than 1 month” and underwent gastroscopy, which revealed GC. On August 8, 2022, she underwent radical gastrectomy (Billroth I anastomosis) under general anesthesia. Her postoperative pathology revealed a small gastric curvature ulcerative mucinous adenocarcinoma, some of which were signet-ring cell carcinoma, measuring 4 x 3.5 cm in size, that had invaded the serous membrane. Moreover, a cancer embolus was detected in the vessel, although no cancer was detected in the upper and lower stumps. (Group 1, group 4, group 5, group 6, group 7/8/9) Lymph nodes were detected with metastatic cancer, respectively (1/1, 7/9, 3/3, 2/2, 3/10); (group 3) fibrous adipose tissues with adenocarcinoma invasion; (group 11) no metastatic carcinoma in lymph nodes (0/1); (group 12) fibrous adipose tissues; no tumor was detected in the omentum. Immunohistochemical results revealed MLH1 (+), MSH2 (+), MSH6 (+), PMS2 (+), Villin (+), Her-2 (-), ER (-), GATA3 (-), and E-cad (-). SOX regimen for 2 cycles was administered after surgery, although the specific dose remains unknown. On July 4, 2023, the examination indicated peritoneal, left ovarian, and multiple bone metastases. Shortly thereafter, the patient passed away on July 11, 2023, with an overall survival (OS) period of over 11 months. She had a history of “right breast cancer,” but the details remain unknown.

## Discussion

HDGC is an autosomal dominant syndrome characterized by a high incidence of diffuse GC and lobular breast cancer (LBC). It is mostly caused by an inactivating mutation of the tumor suppressor gene *CDH1* and a small proportion of HDGC families displaying an abnormal *CTNNA1* (3). The frequency of *CDH1* mutation in Chinese HDGC patients has not yet been reported, although the frequency of *CDH1* mutation in Chinese GC patients has been reported to be <1% (9). The clinicopathological features of HDGC were as follows: (1) incomplete penetrance of autosomal dominant inheritance, with 70–80% penetrance of mutant genes; (2) the onset age is early, aged 14–84 (average: 38) years, with the characteristics of early onset, which maybe 5–6 years earlier than the previous generation; (3) tumor differentiation was poor: the pathological types were mainly poorly differentiated adenocarcinoma and signet ring cell carcinoma, and the Lauren classification was mostly of the diffuse type; (4) early endoscopic diagnosis is difficult: submucosal infiltration and scattered distribution. It is often difficult to obtain tumor tissues under gastroscopy, and random and multi-point sampling is required along with the need for pathological examination; (5) multiple extragastric tumors: colon cancer and female breast cancer are the most common, and other extragastric tumors include esophageal cancer, liver cancer, lung cancer, leukemia, endometrial cancer, and prostate cancer (3). The cumulative lifetime risk of GC in carriers of *CDH1* germline pathogenic or possibly pathogenic mutations is approximately 37–42% in men and 25–33% in women (10). The cumulative risk for GC by age 80 years has been reported to be 56% for women and 70% for men (11). Prophylactic total gastrectomy (PTG) has been recommended by most guidelines. Generally, prophylactic surgery is recommended for patients aged 20–30 years or for the least affected family member of age <5 years (4, 12); however, the complications of this procedure are significant and may adversely affect the patient’s quality of life (13, 14). In addition, there are no preventive medications for *CDH1* carriers (3).

Presently, there is no reliable screening method for the early diagnosis of DGC in mutation carriers. GC in patients with HDGC is signet-ring cell carcinoma, which is located below the intact surface epithelium and can only be detected through direct mucosal assessment at an advanced stage of the disease. Endoscopic surveillance is generally performed by experienced medical centers; the optimal frequency of surveillance has not yet been unified, and it is recommended to be performed once a year (4). In addition, multiple random biopsies are recommended to improve the detection rates (11). The indications for endoscopy are as follows: (1) age <20 years; (2) refusal for surgical resection; (3) the detected mutations were not pathogenic or of unknown significance. The endoscopy was performed with a white light and high-resolution endoscopy for at least 30 min; the mucosa was thoroughly cleaned with a mucolytic agent (N-acetylcysteine) combined with defoamer (simethicone) mixed with sterile water before the examination, and the mucosa during repeated aeration and deaeration was carefully observed, and the biopsy specimens were collected. Image acquisition of all abnormal areas, if the stomach cannot be expanded during aeration, submucosal infiltrating lesions, such as leather stomach, should be suspected. Multiple random biopsies were performed to improve the detection rate. It was accordingly recommended to take at least 30 biopsy specimens from the prepyloric region, gastric antrum, transitional zone, gastric body, gastric fundus, and cardia, 5 from each region. Sampling can be performed with the use of standard biopsy forceps or, preferably, with needle-biopsy forceps, in order to obtain a specimen of the lamina propria (3).

For *CDH1-*mutation carriers, when signet-ring cell carcinoma or diffuse GC is confirmed via gastroscopy, radical total gastrectomy, and perioperative treatment are recommended according to the disease stage (3). If the diagnosis is advanced, systematic drug therapy should follow the existing guidelines for GC (3), and the overall 5-year survival rate of patients with advanced HDGC is only 4% (15). Notably, HDGC, such as several sporadic diffuse types of GC, rarely have the overexpression of human epidermal growth factor receptor-2 (HER-2), which has a low tumor mutational burden and often lacks relevant markers that suggest effective immunotherapy (15). Presently, targeted therapy, immunotherapy, and cell therapy for *CDH1* and its related pathways are at the research stage, and patients are encouraged to actively participate in relevant clinical research (12).

Prenatal screening, as an important means of effective prevention of genetic diseases (including tumor-related genetic diseases), is relatively mature at present. However, the physical and psychological damage to the mother and the family, as well as the ethical issues raised by therapeutic induction of labor, remain controversial. In recent years, preimplantation genetic diagnosis/screening has enabled genetic testing of embryos before implantation in the uterus, which allows selection of embryos that are normal or do not carry any relevant mutations, which are then transferred to block the passage of tumor-related genetic diseases from the source (16, 17). It is recommended that germline mutation carriers with fertility requirements should consult an experienced reproductive center for eugenics (3).

In addition, due to the higher incidence of LBC among female carriers of *CDH1* pathogenic mutations, annual visits to breast specialists and breast magnetic resonance imaging (MRI) may be considered as a preventive measure (3).

Among the present HDGC patients, the first three family members exhibited a clear *CDH1* mutation, with an early onset, as young as 17 years of age. All four affected family members showed pathological types, including signet ring cell carcinoma. Immunohistochemistry of the proband and her grandmother indicated HER2 negativity. Immunohistochemistry or genetic testing indicated pMMR or MSS, and the grandmother of the proband had a history of breast cancer. Both patients had a relatively short survival period, with the proband experiencing the shortest, of a little over 3 months. The aforementioned information closely aligns with the known clinical and pathological characteristics of HDGC. Research indicates that patients with *CDH1* mutations should undergo preventive total gastrectomy before the detection of GC or the emergence of symptoms, as a manifestation of symptoms typically indicates stage III or IV of the disease, which has been associated with a poor prognosis (15, 18). The proband and his grandmother were diagnosed with GC after exhibiting symptoms at stages III and IV, with survival periods of just over 3 months and 11 months, respectively. In contrast, the proband’s mother and maternal aunt were diagnosed with GC before they even exhibited symptoms, and they are currently surviving for over 2 years with an unexpired OS time; this observation is consistent with the literature reports (15, 18). Notably, the proband’s survival duration was extremely short, just over 3 months, which could be attributed to the young age at onset, late disease stage, high rate of malignancy, and *CDH1* germline mutations, as well as concurrent *TP53* mutations. *TP53* mutations render GC more susceptible to progression to advanced DGC (15), and patients with concurrent *TP53* and *CDH1* deletions develop highly aggressive tumors (15). As a classic tumor suppressor gene, TP53 inhibits tumorigenesis through regulating the cell cycle, promoting DNA repair, and inducing apoptosis (19). TP53 mutation is one of the common molecular events in gastric cancer, influencing the clinicopathological characteristics and prognosis of gastric cancer patients (19). Patients with TP53-mutant gastric cancer often present with adverse clinical features such as poor differentiation, large tumor diameter, advanced TNM stage, and lymph node metastasis. These characteristics are closely associated with the loss of cell cycle control resulting from TP53 mutations (19). The occurrence of TP53 mutations not only affects the clinicopathological features of gastric cancer but is also significantly correlated with patient prognosis (19). OS and disease-free survival (DFS) are significantly shorter in TP53-mutant gastric cancer patients when compared to those with wild-type TP53 (19). This finding may be attributed to the loss of p53 protein function due to TP53 mutation, which abrogates its regulatory role in the tumor cell cycle and apoptosis, thereby enhancing tumor cell proliferation and survival capabilities and promoting malignant progression of gastric cancer. Furthermore, TP53 mutations increase tumor cell resistance to chemotherapy and radiotherapy, particularly by reducing sensitivity to DNA damage, thereby elevating the risk of treatment failure and recurrence (19).

The short survival of the proband in this case is also implicated with the NRG1 gene mutation. Neuregulins (NRGs), comprising four members (NRG1-4), are ligands for the ERBB family of tyrosine kinase receptors (i.e., ERBB3 and ERBB4) (20). As a member of the NRG family, NRG1 binds to its receptors and activates downstream signaling pathways, thereby participating in cell survival, proliferation, differentiation, and invasion in both normal and malignant tissues (20). The overexpression of NRG1 can activate the ERBB3/ERBB2-signaling pathway, promoting cancer cell proliferation, invasion, and metastasis. In solid tumors, NRG1 overexpression may represent an independent indicator of poor prognosis (20). In gastric cancer tissues, NRG1 is overexpressed, which is closely associated with aggressive clinicopathological parameters such as larger tumor size, invasive growth, and lymph node metastasis. Furthermore, NRG1 can activate the NF-κB-signaling pathway to promote the self-renewal of gastric cancer stem cells, indicating that NRG1 overexpression predicts an unfavorable prognosis in gastric cancer patients (20). In summary, the extremely short survival of the proband in this case report may be fundamentally attributed to the concurrent presence of a CHD1 germline mutation along with TP53 and NRG1 gene mutations. However, a comprehensive review of the current literature revealed no similar reported cases.

In addition, past studies have reported that HDGC occurs at an early age and has early-onset characteristics. In such cases, the offspring may develop HDGC several years earlier than in the previous generation (3). Men carrying *CDH1* mutations are more likely to develop HDGC than women (21), and *CDH1* mutations have been significantly associated with poor survival prognosis. Young patients, especially those aged <35 years, have a poorer prognosis (22). In this study, the proband patient was a male who developed HDGC at the age of 17 years and had an earlier onset age than in the previous generation, with a shorter survival period. The other three family members who developed HDGC were all females, with a later onset age and a relatively longer survival period, which is consistent with the literature (3, 21, 22). According to the literature, congenital anomalies such as cleft lip/palate or blepharocheilodontic syndrome are frequently observed in some *CDH1-*mutation carriers (23). In this report, the proband exhibited a congenital absence of the lower incisors, whereas the other three women did not exhibit this particular developmental abnormality. There are no published reports on this congenital developmental abnormality in *CDH1* mutation carriers or HDGC. Further exploration is thus warranted to determine whether there exist any gender-based differences in its genetic association. Based on the treatment histories of the two deceased family members reported herein, it is evident that while advanced HDGC can be treated according to the current GC guidelines, including radical surgery and systemic drug therapy, the outcomes are frequently disappointing, with a poor response to treatment. In addition, often the diagnosis of advanced-stage disease is followed by an extremely poor prognosis and survival. Therefore, long-term follow-up is necessary for family members diagnosed with *CDH1* pathogenic mutations (3). If PTG is not an option, annual gastroscopy is recommended, and first-degree relatives of *CDH1* germline mutation carriers should undergo *CDH1* germline mutation gene screening to facilitate early detection, diagnosis, and treatment, so as to improve the prognosis (3).

**Figure 4:**
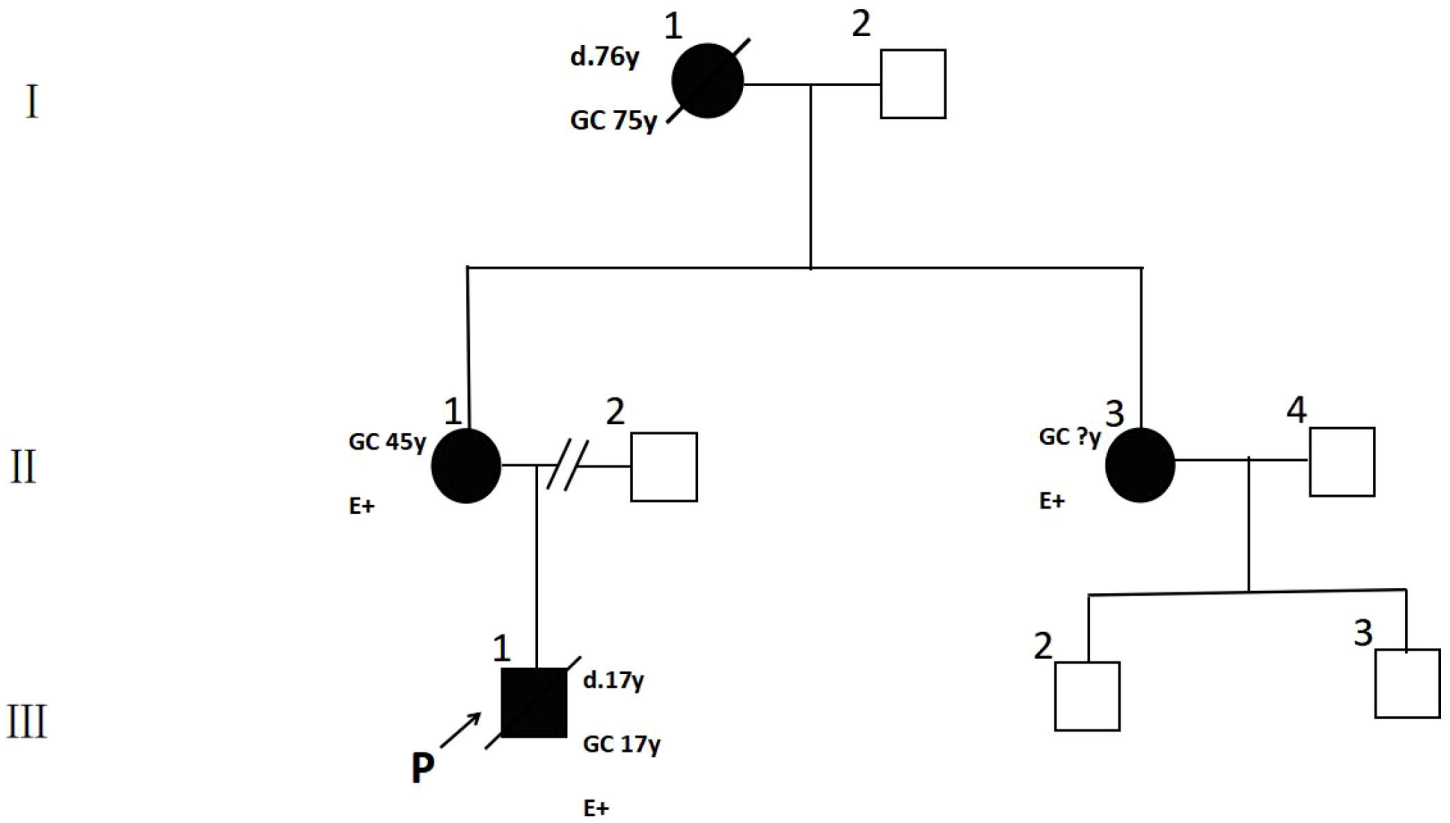
Pedigree of family with HDGC. The shaded square and circle represent the family members with gastric cancer. GC age at diagnosis of gastric cancer; E+ indicated CDH1 mutation positive; E− indicated CDH1 mutation negative, P indicated the proband in this family.

In summary, this study reports a case of HDGC in a Chinese family spanning three generations and involving four members (the proband and their relatives) (**Figure 4**). A comprehensive, multi-dimensional analysis of the pedigree was conducted by systematically collecting clinical history, pathological sections, imaging data (such as CT scans and gastroscopy), genetic testing results, detailed family history, treatment process, and survival outcomes. A pathogenic germline mutation in *CDH1* was identified in this family. The clinical manifestations among different mutation carriers varied significantly; some developed diffuse gastric cancer at an early age, while others exhibited only early-stage lesions or remained asymptomatic, which highlights the complexity of genotype-phenotype correlations. The diagnosis and treatment process fully reflects the challenges in managing HDGC, including the difficult decision-making regarding PTG, the limitations of endoscopic surveillance, the psychological burden faced by patients and their families, and the urgent need for reproductive genetic interventions (such as using preimplantation genetic testing to prevent transmission of the pathogenic variant). This study underscores the critical importance of genetic testing and genetic counseling for gastric cancer patients with a family history and early onset of disease. Personalized risk management strategies based on genetic results, including prophylactic surgery and intensive monitoring, are key to improving prognosis. These findings provide valuable empirical evidence for clinicians managing similar cases and contribute significantly to promoting a multidisciplinary team (MDT) approach, genetic counseling, and reproductive interventions for HDGC.

## Data Availability

The original contributions presented in the study are included in the article/Supplementary Material, further inquiries can be directed to the corresponding author.
